# Understanding silicate hydration from quantitative analyses of hydrating tricalcium silicates

**DOI:** 10.1038/ncomms10952

**Published:** 2016-03-24

**Authors:** Elizaveta Pustovgar, Rahul P. Sangodkar, Andrey S. Andreev, Marta Palacios, Bradley F. Chmelka, Robert J. Flatt, Jean-Baptiste d'Espinose de Lacaillerie

**Affiliations:** 1 Institute for Building Materials, Department of Civil, Environmental and Geomatic Engineering, ETH Zürich 8093, Switzerland; 2 Department of Chemical Engineering, University of California, Santa Barbara, California 93106, USA; 3 Soft Matter Science and Engineering Laboratory, UMR CNRS 7615, ESPCI Paris, PSL Research University, 10 rue Vauquelin, Paris 75005, France

## Abstract

Silicate hydration is prevalent in natural and technological processes, such as, mineral weathering, glass alteration, zeolite syntheses and cement hydration. Tricalcium silicate (Ca_3_SiO_5_), the main constituent of Portland cement, is amongst the most reactive silicates in water. Despite its widespread industrial use, the reaction of Ca_3_SiO_5_ with water to form calcium-silicate-hydrates (C-S-H) still hosts many open questions. Here, we show that solid-state nuclear magnetic resonance measurements of ^29^Si-enriched triclinic Ca_3_SiO_5_ enable the quantitative monitoring of the hydration process in terms of transient local molecular composition, extent of silicate hydration and polymerization. This provides insights on the relative influence of surface hydroxylation and hydrate precipitation on the hydration rate. When the rate drops, the amount of hydroxylated Ca_3_SiO_5_ decreases, thus demonstrating the partial passivation of the surface during the deceleration stage. Moreover, the relative quantities of monomers, dimers, pentamers and octamers in the C-S-H structure are measured.

## Abstract (web summary)

Despite its importance to the cement industry, tricalcium silicate hydration, with its sequence of induction, acceleration and deceleration steps, still hosts many open questions. Here, ^29^Si nuclear magnetic resonance provides new information on the silicate polymerization mechanism and its kinetics.

## Graphical abstract

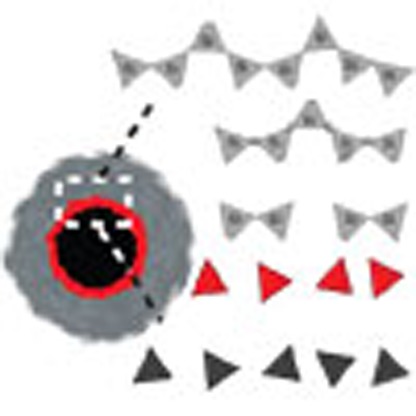

Since Le Chatelier^1^, it is well understood that Portland cement hydration is initiated by the dissolution of calcium silicate monomers in water, followed by the precipitation of less soluble layered calcium-silicate-hydrates (C-S-H), in which silicate ions condense to form short chains. However, despite two centuries of widespread applications and a century of detailed study, the molecular mechanisms behind the kinetic stages of hydration (that is, induction, acceleration and deceleration) are still debated. Similar kinetic stages are observed in various heterogeneous hydration processes occurring during mineral weathering^2^,^3^, glass alteration^4^,^5^ and hydrothermal syntheses. For example, although hydrothermal zeolite syntheses under alkaline aqueous conditions proceeds over different timescales^6^, the effective reaction rates in cementitious and zeolite systems exhibit similar distinct stages (induction, acceleration and deceleration), and are governed by several coupled parameters varying in space and time near the liquid–solid interface. This situation is thus extremely complex to describe accurately. An added difficulty is that for porous materials such as cement or zeolites, interfacial energy contributes to the stabilization of nanoscale intermediates, which are typically challenging to characterize. For Portland cement in particular, the lack of quantitative experimental data obtained with sufficient time resolution has precluded the validation of existing models aimed at explaining the complex kinetics of cement hydration.

Similar to the homogeneous versus heterogeneous pathways dichotomy in zeolite crystallization mechanisms^7^, two landmark competing theories have been proposed to explain the early-age time dependence of the rate of tricalcium silicate (Ca_3_SiO_5_) hydration, the principal component in commercial Portland cements responsible for the development of mechanical strength^8^,^9^,^10^. The first theory proposes that early-age hydration products form a diffusion barrier on the surfaces of Ca_3_SiO_5_ particles, thus affecting subsequent reactions of the underlying non-hydrated core^11^. The second theory^12^,^13^,^14^ suggests that the early-age time-dependence of the rate of hydration is determined by the rate of Ca_3_SiO_5_ dissolution and by a change in the associated rate limiting step from etch pit formation to step retreat, which is a mechanism also often invoked in the geochemical literature on natural weathering^15^,^16^. The relevance of these theories to silicate hydration can be examined by understanding the molecular compositions and structures of species at the solid–liquid interfaces during the early stages of hydration. Similar questions are raised in heterogeneous catalysis and geochemistry; however, Portland cement hydration faces the additional complexity that the main product, C-S-H, is not only poorly crystalline but also nanostructured with variable stoichiometry and silicate coordinations^17^,^18^. These challenges have been previously addressed partially through numerical modelling of hydration reactions at Ca_3_SiO_5_ surfaces^19^,^20^ and of the local structure and disorder of the resulting hydration products^21^. Nevertheless, these models suffer from a lack of experimental support at the molecular level.

Here, solid-state NMR measurements of triclinic ^29^Si-enriched Ca_3_SiO_5_ hydration are used to determine the transient molecular-level compositions at silicate surfaces and the interactions between silicate species, hydroxyl groups and water molecules, which influence the rates of hydration reactions. The isotopic ^29^Si enrichment provides significantly enhanced NMR signal sensitivity that can be used to monitor the structures of the hydrates *in situ* during the hydration process, as a function of hydration time. In addition, ^29^Si enrichment enabled two-dimensional (2D) through-bond (*J*-mediated) NMR measurements that are sensitive to ^29^Si-O-^29^Si covalent bonding. They are used to crucially provide detailed information on the local atomic-level compositions, structures and site connectivities in hydrated silicate species, here C-S-H. These analyses shed new insights on the origin of rate limiting steps and the kinetics of silicate polymerization at the solid–liquid interface during Ca_3_SiO_5_ hydration.

## Results

### Experimental approach

**Figure 1:**
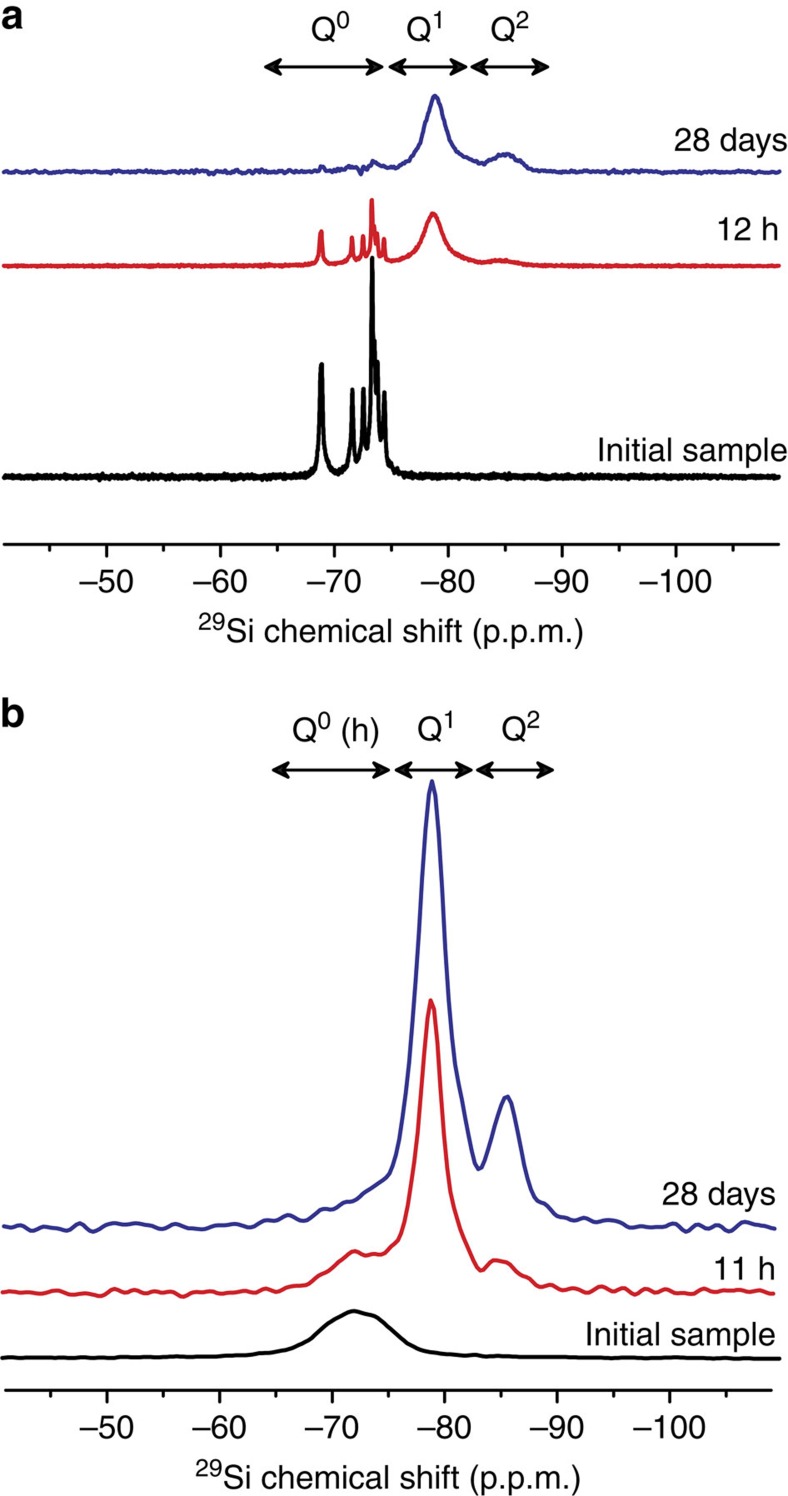
Dynamics of silicate hydrates formation studied *in situ* by ^29^Si NMR. (**a**) ^29^Si MAS NMR and (**b**) {^1^H}^29^Si CPMAS NMR spectra of ^29^Si-enriched triclinic Ca_3_SiO_5_ sample in its initial non-hydrated state (in black) and after hydration for 11 or 12 h (in red) and 28 days (in blue). ^29^Si resonances from isolated silicate (Q^0^) species in non-hydrated Ca_3_SiO_5_, hydoxylated surface Q^0^ (Q^0^(h)) species and polymerized calcium-silicate-hydrates (Q^1^ and Q^2^) are clearly resolved and can be quantified as a function of time.

To the seminal approach of ^29^Si enrichment by Brough *et al*.^22^, we added for the first time the sophistication of carefully controlled structure and granulometry of the Ca_3_SiO_5_ particles (see Supplementary Methods) and hydration reaction conditions (see Supplementary Notes 1 and 2). Indeed the surface structure and area of the Ca_3_SiO_5_ particles strongly affect their reactivity, which must be carefully controlled to ensure meaningful results^23^. For example, the high surface area of the synthesized ^29^Si-enriched Ca_3_SiO_5_ (4.4 m^2^ g^−1^, see Supplementary Methods) allowed ∼90% of the silicate hydration process to be monitored in 24 h of NMR spectrometer time, without external acceleration. In this way, subtle and unique quantitative information pertinent to hydration mechanisms can be obtained non-invasively and with a time resolution of 30 min (measurement time for the NMR spectra). Consequently, the progress of the hydration reaction could be accurately and quantitatively correlated to the corresponding ^29^Si speciation. In addition, ^29^Si enrichment allows NMR measurements to be performed on samples without the need for conventional water removal schemes for quenching the hydration process^24^, which otherwise often disrupt the fragile microstructure of the C-S-H or may detrimentally alter chemical composition. Representative one-pulse ^29^Si and ^1^H{^29^Si} cross-polarization (CP) magic-angle-spinning (MAS) NMR spectra are presented in Fig. 1a,b, respectively, for non-hydrated and hydrated Ca_3_SiO_5_. In anhydrous triclinic Ca_3_SiO_5_ which exhibits long-range crystalline order and well-defined local atomic ^29^Si environments, eight distinct and narrow (<0.5 p.p.m. full-width at half maximum (FWHM)) ^29^Si signals are resolved between −68 and −75 p.p.m. corresponding to anhydrous Q^0^ species (Supplementary Fig. 2). In contrast, in hydration products, the ^29^Si resonances are broad (3–4 p.p.m. FWHM) with signals centred at −72, −79 and −85 p.p.m. from silanol Q^0^(h), hydrated Q^1^ and hydrated Q^2^ silicate species, respectively (Fig. 1). The last two species are associated with the C-S-H structure (Q^*n*^ refers to silicon atoms that are covalently bonded via bridging oxygen atoms to 0≤*n*≤4 other silicon atoms^25^). These molecular-level insights of the local silicate structures in Ca_3_SiO_5_ hydration products (C-S-H) are consistent with previous ^29^Si NMR (refs 26, 27), ^17^O NMR (ref. 28), X-ray and neutron scattering results^18^ for C-S-H.

**Figure 2:**
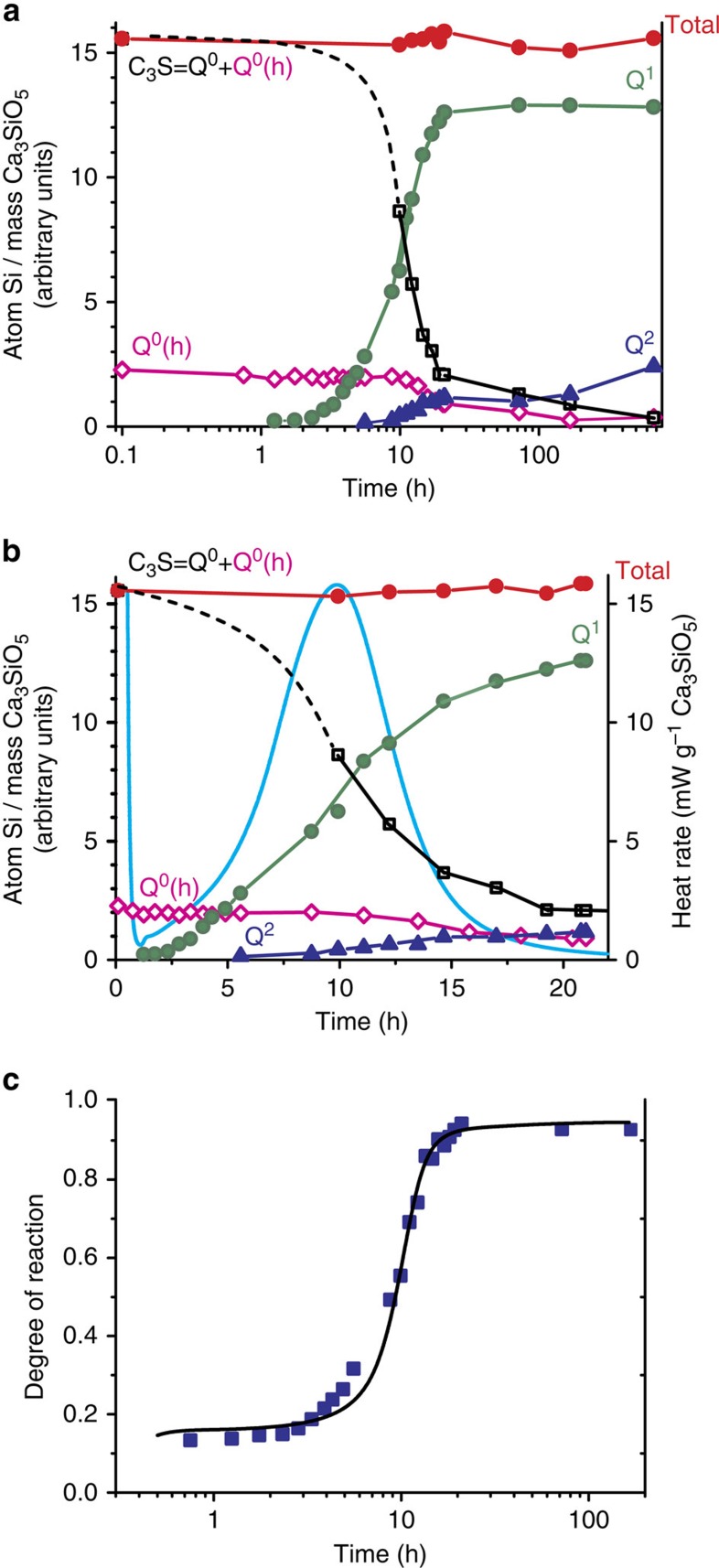
Quantitative monitoring of silicate speciation during the hydration of ^29^Si-enriched triclinic Ca_3_SiO_5_. (**a**) The quantities of different ^29^Si silicate species as established by ^29^Si MAS and {^1^H}^29^Si CPMAS NMR measurements for hydration times up to 28 days (see Supplementary Note 1). The quantities, normalized to the initial amount of Ca_3_SiO_5_, of anhydrous Q^0^ (in black), hydroxylated Q^0^(h) (in pink), hydrated Q^1^ (in green), hydrated Q^2^ (in blue) and total silicate species (in red) resulting from this analysis are as shown. (**b**) Comparison of the quantities of different ^29^Si silicate species and the reaction heat flow rate determined by isothermal calorimetry (cyan line) for Ca_3_SiO_5_ up to 24 h of hydration. Based on the heat released in the calorimetry measurements, four stages in the hydration process can be identified: first a brief exothermic peak during the first few minutes (<15 min) corresponding to initial dissolution of Ca_3_SiO_5_, then a short (15 min–2 h) induction period during which no significant heat is released, followed by a peak corresponding to the acceleration period (2–10 h), and finally the deceleration period (>10 h) associated with decreasing rate of heat release (Supplementary Fig. 8). (**c**) Comparison of the degree of silicate hydration determined independently by ^29^Si MAS and {^1^H}^29^Si CPMAS NMR quantitative analyses (squares) and isothermal calorimetry results (black line), which are in close agreement. The fact that the total amount of Si atoms remains constant, within the uncertainties of the measurements, over the entire hydration period (28 days) establishes the accuracy of the associated quantitative NMR methods and analyses. Details of these analyses are included in the Supplementary Note 1.

The degree of silicate hydration is determined by quantitative *in situ*^29^Si NMR analyses and forms the crux of our results, which are summarized in Fig. 2. These results are in close agreement with the degree of silicate hydration as established by independent isothermal calorimetric measurements, which reveal the successive stages of initial dissolution, induction, acceleration and deceleration (Fig. 2b) during the silicate hydration process. This comparison crucially establishes the accuracy of the quantitative ^29^Si NMR results acquired during Ca_3_SiO_5_ hydration, and indicates that the hydration process is negligibly altered by factors such as the MAS conditions of the NMR experiment (see Supplementary Notes 1 and 2). This detailed time-resolved, *in situ*, quantitative NMR analysis answers three central questions about Ca_3_SiO_5_ hydration: the molecular origin of the reduced apparent solubility of Ca_3_SiO_5_ during the induction period, the possible ‘switch' from one type of hydration products to another between the acceleration and deceleration period, and the relative proportions of silica oligomers in the final C-S-H structure.

### Induction period

**Figure 3:**
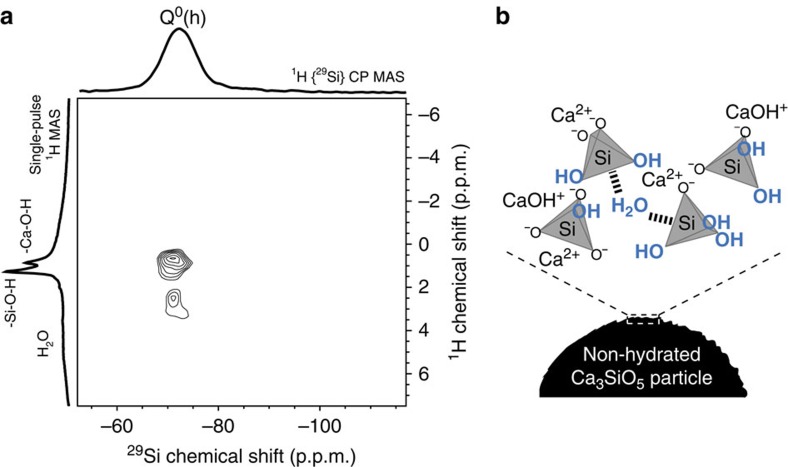
Proton to silicon signal intensity correlations on the initial non-hydrated ^29^Si-enriched triclinic Ca_3_SiO_5_. (**a**) The 2D {^1^H} ^29^Si HETCOR NMR spectrum shows intensity correlations between ^29^Si and ^1^H signals that result from molecular proximity between ^29^Si and ^1^H nuclei. ^29^Si CPMAS and ^1^H MAS 1D spectra are shown along the horizontal and vertical axis of the 2D spectrum. The chemical shift of ^29^Si is detected (horizontal dimension), while chemical shift of ^1^H is recorded in the indirect (vertical) dimension. (**b**) The right inset schematizes the protonated moieties detected on the Ca_3_SiO_5_ surfaces.

The apparent solubility of Ca_3_SiO_5_ during the induction period of hydration has been reported to be lower compared with pristine anhydrous Ca_3_SiO_5_ (refs 11, 12, 13). This reduced apparent solubility has been proposed to arise from the deposition of a layer of hydration products (the metastable barrier hypothesis)^11^ or from surface hydroxylation^12^,^13^. The molecular compositions at the Ca_3_SiO_5_ surface during this induction period (as determined by the NMR analyses presented here) points towards the latter scenario. The ^29^Si{^1^H} CPMAS NMR measurements of the initial sample (that is, non-hydrated) (Fig. 1b) establish the presence of Q^0^ silicate species in proximity to protons (henceforth labelled Q^0^(h)) on Ca_3_SiO_5_ particle surfaces, even before contact with bulk water. Although previous studies have reported the presence of similar Q^0^(h) silicate species at the surfaces of ‘anhydrous' Ca_3_SiO_5_ particles^22^,^26^, it has not been largely publicized nor quantitatively analysed. The 2D ^29^Si{^1^H} heteronuclear correlation (HETCOR) NMR spectrum of the same sample of non-hydrated Ca_3_SiO_5_ (Fig. 3) exhibits correlated intensities between the ^29^Si signal at −72 p.p.m. from Q^0^(h) species and unresolved ^1^H signals around 1.3 and 0.9 p.p.m. from –SiOH and -CaOH moieties, thereby establishing the close molecular-level proximities of surface Q^0^(h) species to at least one type of such ^1^H moieties. In addition, the absence of resonances characteristic of polymerized hydration products (that is, Q^1^ and Q^2^ species), establishes that the reaction of surface silicate species in non-hydrated Ca_3_SiO_5_ with atmospheric moisture results solely in the formation of hydroxylated Q^0^(h) species at particle surfaces, within the sensitivity limits of the measurement. In other words, no separate hydrate phase forms at this stage, it is solely the Ca_3_SiO_5_ particle near-surface which is hydroxylated.

From a crystal chemistry perspective, the Ca_3_SiO_5_ particle surface is unlikely to be inert when exposed to atmospheric water vapour. Specifically, Ca_3_SiO_5_ is an ionic crystal of Ca^2+^ cations with oxide and monomeric silicate anions (3Ca^2+^·O^2−^·SiO_4_^4−^) (refs 19, 29). There is a strong ionization of the atoms (+1.5 on Ca^2+^ and −1.5 on O^2−^) (ref. 19) and consequently Ca_3_SiO_5_ acts as a basic oxide that readily yields hydroxide ions when reacting with water,


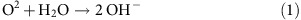


Therefore, one expects OH^−^ to replace oxide ions on the particle surfaces. However, replacement of one O^2−^ by two OH^−^ would yield a heterogeneous distribution of local atomic environments at the Ca_3_SiO_5_ surface, due to the different sizes and formal charges of these anions. Indeed the Q^0^(h) ^29^Si NMR resonance of the initial sample is very broad (Fig. 1b), reflecting a wide distribution of local ^29^Si environments. In summary, the ^29^Si NMR analyses reveal that near-surface ^29^Si species on Ca_3_SiO_5_ particles are predominantly hydroxylated and that negligible quantities of polymerized silicate hydration products form (within the sensitive detection limits of the measurements), a result consistent with previous force-field atomistic simulations^19^. Overall, hydroxylated Q^0^ (h) species are predominant at particle surfaces during the induction period and expected to result in the reduced apparent solubility of Ca_3_SiO_5_, compared with pristine anhydrous Ca_3_SiO_5_ whose level of hydroxylation is lower.

### Acceleration stage

With the progress of Ca_3_SiO_5_ hydration, the monomeric Q^0^ silicate species polymerize to form oligomeric units of C-S-H. As shown in Fig. 2, while the population of hydroxylated Q^0^(h) species remains constant, the populations of Q^1^ species increase significantly during the acceleration stage (∼2–10 h). Compared with the induction stage (<2 h), the ^29^Si polymerization during the acceleration stage results predominantly in the formation of Q^1^ species (dimers) at early times, and a combination of Q^1^ and Q^2^ species (for example, pentamers and octamers) at later time (10–20 h). In particular, the population of Q^1^ species increases approximately linearly with the progress of hydration (Fig. 2b) across the entire acceleration stage, consistent with the formation of predominantly dimeric C-S-H units. No significant change nor in the silicon second coordination sphere of the hydration products nor in their rate of formation could be detected at this stage.

### Deceleration stage

The data in Fig. 2 indicate that at the end of the acceleration stage (after ∼10 h in the present case) greater quantities of long (>2 silicate tetrahedra) C-S-H chains containing Q^2^ species are formed compared with dimeric C-S-H units (without Q^2^). Although the amounts of Q^2^ species increase progressively after the hydration peak (∼20 h), the population of Q^1^ species remains approximately constant, which indicates the formation of longer silicate chains besides the dimers. By comparison, the amount of Q^0^(h) species remains constant for several hours (∼10 h) during the induction and acceleration stages, it subsequently decreases just when, according to isothermal calorimetry, the Ca_3_SiO_5_ hydration slows down, that is during the so-called deceleration stage. This observation provides important insights regarding the debate on the origin of the deceleration period. While some previous studies suggest that the deceleration period results from coverage of Ca_3_SiO_5_ particles by hydration products^30^, others claim that hydration initially results in products forming a low-density structure, the subsequent densification of which corresponds to the beginning of the deceleration stage^31^,^32^. Our analyses suggest that compared with the acceleration period that is associated with the formation of predominantly dimeric C-S-H units, the deceleration period corresponds to the formation of greater relative fractions of C-S-H units with longer chain lengths. Such increasing extents of silicate polymerization might possibly be accompanied by an increased density of the C-S-H that consequently would present a diffusion barrier for mass transport and, thus, slow the rate of hydration reaction, consistent with the deceleration stage. This alone is not conclusive as it could either support the view according to which the deceleration would be based indeed on the filling of an ultra-low-density gel^33^ or the one based on an inhibition of hydration by hydrates themselves^34^, impinging on each other's growth^35^,^36^. Nevertheless, the decrease of the amount of near-surface Q^0^(h) species population at the onset of the deceleration period reflects a proportional decrease of the average surface area available to drive hydration by silicate dissolution. The decrease of the particles surface area as revealed here by NMR supports strongly the conclusions of recent modelling studies^37^, namely that the deceleration stage results from the reduction of the average particle surface area available for reaction due to increasing surface coverage of the Ca_3_SiO_5_ particles by hydration products. This conclusion is also supported by the fact that at 7 days 5% of the Ca_3_SiO_5_ has not yet hydrated, bringing support to a coverage and passivation of its surface by deposited hydrates. Moreover, the long period during which Q^0^(h) remains constant suggests that during dissolution, the surface decrease due to the reduction in particle size is compensated by roughening (opening of etch pits and step retreat)^38^. In other words dissolution does not simply proceed by shrinking of the core of the particles, but also by etching.

### Final C-S-H structure

**Figure 4:**
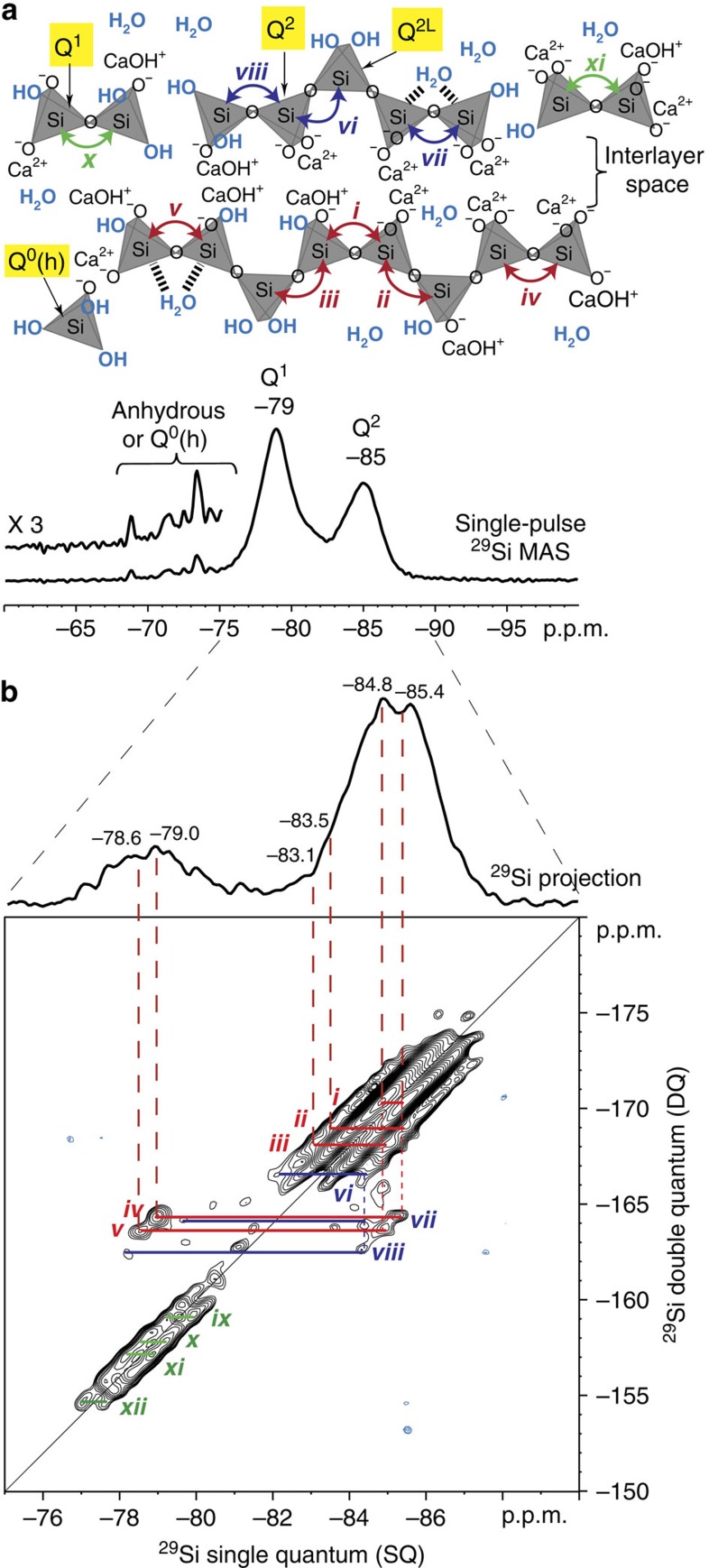
Molecular structures and silicate site connectivities in partially polymerized calcium-silicate-hydrates. (**a**,**b**) Solid-state (**a**) 1D single-pulse ^29^Si MAS and (**b**) 2D *J*-mediated ^29^Si{^29^Si} correlation NMR spectra of hydrated (1.5 month, 25 °C) ^29^Si-enriched triclinic Ca_3_SiO_5_. The lowest contour lines in the 2D spectrum are 9% of the maximum signal intensity. The ‘double-quantum' filter used to acquire the spectrum in **b** enables selective detection of pairs of signals (*i*, *j*) from distinct ^29^Si nuclei that are covalently bonded. Consequently, the 2D spectrum exhibits intensity correlations between ^29^Si signals at distinct frequencies (*ω*_*i*_, *ω*_*j*_) from ^29^Si-O-^29^Si spin pairs (*i*, *j*) in the horizontal SQ dimension (isotropic ^29^Si chemical shifts) and at the sum of these frequencies (*ω*_*i*_+*ω*_*j*_) in the vertical DQ dimension. Therefore, correlated intensities at these specific positions in the 2D spectrum unambiguously establish the presence of covalently bonded ^29^Si silicate species corresponding to the distinct isotropic ^29^Si chemical shifts. The inset in **a** shows a schematic diagram of the different silicate moieties present in the calcium-silicate-hydrates with double-headed arrows indicating the *J*-interactions in ^29^Si-O-^29^Si species that are established by the intensity correlations in the 2D spectrum, specifically from dimeric (green), pentameric (blue) or octameric (red) units. For sake of clarity, the calcium layers are not represented.

The atomic site interconnectivities of different silicate species can be used to elucidate the molecular structures and lengths of silicate chains in the C-S-H. Such detailed insights can be obtained by using solid-state 2D *J*-mediated ^29^Si{^29^Si} correlation NMR techniques^39^ that probe *J*-coupled ^29^Si-O-^29^Si spin pairs and have been previously applied to establish silicate framework connectivities in a variety of heterogeneous materials^40^,^41^,^42^,^43^. Previously, Brunet *et al*.^44^ have conducted 2D dipolar-mediated ^29^Si{^29^Si} NMR measurements that rely on through-space ^29^Si-^29^Si dipolar couplings and which yield information on the molecular-level proximities of different ^29^Si moieties in synthetic C-S-H. However, such measurements cannot be used to directly establish the covalent connectivity among different ^29^Si moieties in the C-S-H structure. In contrast, by relying on through-bond *J*-interactions associated with ^29^Si-O-^29^Si moieties (*J*-interactions between ^29^Si spin pairs separated by more than two covalent bonds are negligibly small and consequently expected to be below the detection limits of the 2D *J*-mediated ^29^Si{^29^Si} NMR measurement.), 2D *J*-mediated ^29^Si{^29^Si} double-quantum (DQ) correlation NMR measurements provide detailed insights regarding the tetrahedral site connectivity in the C-S-H chains. Notably, the 2D *J*-mediated ^29^Si{^29^Si} NMR spectrum of hydrated ^29^Si-labelled Ca_3_SiO_5_ shown in Fig. 4b provides significantly enhanced ^29^Si resolution, compared with the single-pulse ^29^Si MAS spectrum (Fig. 4a), and unambiguously establishes distinct ^29^Si-O-^29^Si covalent connectivities in the silicate chains.

The 2D *J*-mediated ^29^Si{^29^Si} NMR spectrum (Fig. 4b) exhibits three well separated regions of correlated intensities in the Q^1^ (approximately −79 p.p.m.) and Q^2^ (approximately −85 p.p.m.) chemical shift ranges along the single-quantum (SQ)–DQ *y*=2*x* line, and two pairs of cross-correlated peaks between the Q^1^ and Q^2^ chemical shifts ranges. The broad continuous distribution of correlated chemical shifts in the 2D ^29^Si{^29^Si} spectrum between signals at −82 and −87 p.p.m. in the ^29^Si SQ dimension are attributed to different ^29^Si-O-^29^Si Q^2^ moieties, consistent with the structural disorder of C-S-H. Interestingly, the spectrum reveals narrow (0.6 p.p.m. FWHM) ridges of intensity correlations that are parallel to the SQ–DQ line. Such features typically arise from structural disorder on length scales (>1 nm) that are larger than the distances between the ^29^Si-^29^Si spin pairs (or also due to anisotropy in the magnetic susceptibility)^45^. The presence of such poor long-range structural order is consistent with the broad distributions of local ^29^Si environments that are associated with the heterogeneous nature of the C-S-H. Nevertheless, careful analysis of the 2D spectrum distinguishes discrete correlated signal intensities that are resolved to greater than a tenth of a p.p.m. Specifically, a strong correlated intensity (labelled *i*) between the ^29^Si signals centred at −84.8 and −85.4 p.p.m. in the SQ dimension and at −170.2 p.p.m. in the DQ dimension (Supplementary Fig. 11) unambiguously establishes the presence of two chemically distinct Q^2 29^Si species that are covalently bonded through a shared bridging oxygen atom. The different isotropic ^29^Si chemical shifts of these distinct Q^2^ species likely reflect differences in the number and types of species in the C-S-H interlayer (calcium ions or proton moieties such OH groups or water molecules) that are in close (<1 nm) molecular-level proximity to the non-bridging oxygen atoms of the four-coordinate silicate units. Indeed, the different electronegativities of Ca^2+^ and H^+^ result in different ^29^Si nuclear shielding, as shown by recent density functional theory calculations^46^. These molecular-level differences in the Q^2^ species are shown in the schematic diagram (Fig. 4, inset) of a postulated structure of C-S-H that is consistent with the observed 2D NMR correlations (as well as previous experimental^28^,^18^ and modelling analyses^17^,^47^). Although the Q^2L^ resonances (the four-coordinate Q^2^ silicate units that are positioned away from the interlayer space between two C-S-H chains, as shown in the inset in Fig. 4) are not resolved in the spectrum, the external ridges of the Q^2^ correlation spot correspond to correlated intensity between the ^29^Si SQ signals of the two Q^2^ silicate species at -85.4 and −84.8 p.p.m. with the ^29^Si SQ signals from the Q^2L^ species to which they are, respectively, bound. Within this hypothesis and with the constraint that the DQ frequency must be the sum of the SQ frequencies, two additional correlations can be identified for the Q^2^ species at SQ signals -85.4 and −84.8 p.p.m. at DQ signals approximately −168.9 p.p.m. (*ii*) and −168.1 p.p.m. (*iii*), respectively, thus establishing the presence of two distinct Q^2L^ species with SQ signals at -83.5 and −83.1 p.p.m. Furthermore, the same ^29^Si SQ signals at −85.4 and −84.8 p.p.m. from the two Q^2^ silicate species are also separately correlated with ^29^Si signals centred around -79 p.p.m. (*iv*, *v*) (DQ ≃ −164 p.p.m.) from Q^1^ species, further corroborating that these Q^2^ species are indeed chemically distinct. Therefore, analyses of the 2D *J*-mediated ^29^Si{^29^Si} spectrum establish the occurrence of oligomeric silicate units with two distinct Q^2^ and two distinct Q^2L^ species in the C-S-H structure.

The partially resolved pair correlated intensities (*ix*–*xii*) in the range of −77 to −80 p.p.m. reveal the presence of different types of Q^1^ silicate species associated with at least four distinct dimeric C-S-H units. These results are further corroborated by differences in the spin–spin (*T*_2_) relaxation-time behaviours of the associated ^29^Si Q^1^ species, which were exploited to provide improved ^29^Si resolution by using one-dimensional (1D) *T*_2_-filtered ^29^Si MAS measurements (Supplementary Fig. 12). In combination, the different pair correlated intensities establish the presence of dimeric units (*ix*–*xii*) and C-S-H chains that consist of two distinct Q^1^-Q^2^ (*iv*, *v*) and Q^2^-Q^2L^ (*ii, iii)* connectivities and at least one Q^2^-Q^2^ (*i*) connectivity. To accommodate this diversity of atomic connectivity revealed by the 2D ^29^Si{^29^Si} NMR measurements, the C-S-H structure must contain a linear chain of at least eight four-coordinated silicate units (that is, an octamer). A similar analysis of pair correlated intensities *vi*–*viii* indicate the presence of pentameric C-S-H units, as discussed in the Supplementary Note 4. This result is supported by recent studies using density functional theory that have evaluated the relative stabilities of linear C-S-H units of different chain lengths and proposed the presence of stable octameric units^48^, for which no direct experimental evidence has previously been available.

**Figure 5:**
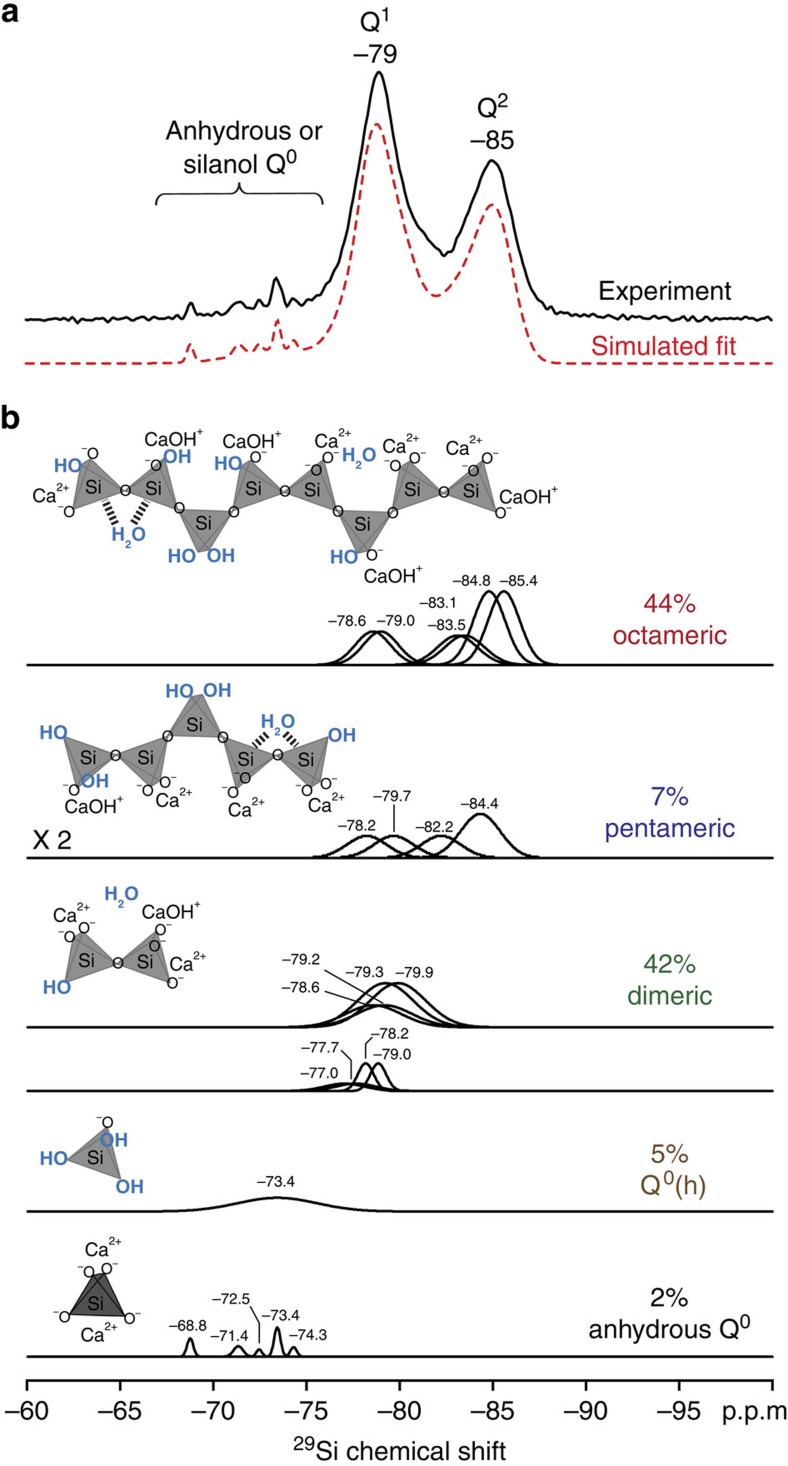
Relative populations of ^29^Si silicate species in hydrated triclinic Ca_3_SiO_5_. (**a**) Solid-state 1D single-pulse ^29^Si MAS spectrum (black) of hydrated (1.5 month, 25 °C) ^29^Si-enriched Ca_3_SiO_5_ and corresponding simulated fit (red) to the spectrum based on the signal decompositions shown in **b**. (**b**) Signal decompositions and relative populations of the different ^29^Si moieties that comprise anhydrous Q^0^, Q^0^(h) and octameric, pentameric and dimeric C-S-H units, which contribute to the simulated fit (red) in **a**. Insets in **b** show schematic diagrams of the possible types of C-S-H units and the associated silicate moieties.

The relative populations of ^29^Si silicate species associated with C-S-H units of different chain lengths (for example, dimers and octamers) are determined based on the enhanced ^29^Si resolution afforded by the 2D ^29^Si{^29^Si} NMR spectrum. Specifically, the single-pulse ^29^Si MAS spectrum shown in Fig. 5a can be simulated by using the peak positions of ^29^Si signals as established by the 2D ^29^Si{^29^Si} NMR spectrum and the relative fractions of Q^1^, Q^2^ and Q^2L^ species associated with C-S-H units of different chain lengths (for example, Q^2^/Q^1^=2, Q^2^/Q^2L^=2 for octamer as shown in Fig. 5b). Such an analysis yields estimates of 44, 7 and 42% (± 4%) for the relative populations of ^29^Si silicate engaged in octameric, pentameric and dimeric units, respectively. These values correspond to 20 mole% octamers, 5 mole% pentamers and 75 mole% dimers in the C-S-H. The salient result is, thus, that despite the fact that the average chain length is 5, pentamers are actually a minority feature. Such distributions of chain lengths are consistent with previous studies that have reported mean chain lengths for C-S-H, which suggest the presence of pentamers and octamers, in addition to dimers^22^,^49^,^50^,^51^. It must be understood that the high amount of octamers was obtained here in a relatively short hydration times (1.5 month) compared with what would be required in a usual cement paste. Specifically, the use of pure tricalcium silicate, the high surface area (4.4 m^2^ g^−1^) of the non-hydrated sample and the water-to-solids ratio (0.8) used in this study are expected to result in relatively fast hydration kinetics and a faster precipitation of C-S-H. The end result is a higher extent of hydration and silicate cross-linking. Interestingly, the analysis also indicates that small quantities of monomeric ^29^Si silicate species, such as hydroxylated Q^0^(h) (5±1%) and anhydrous Q^0^ (2±1%), are present even after hydration of Ca_3_SiO_5_ for 1.5 months at 25 °C. These monomers likely arise from remnants of surface hydroxylation of Ca_3_SiO_5_ particles or are components of the C-S-H structure, which is consistent with recent numerical modelling results^47^.

## Discussion

The carefully synthesized ^29^Si-enriched sample enables, for the first time, 2D *J*-mediated (through ^29^Si-O-^29^Si bonds) ^29^Si{^29^Si} NMR measurements that provide detailed insights regarding the different silicate species, their respective site connectivities, and relative populations, especially for previously unidentified discrete silicate moieties in the C-S-H. Consequently, the lengths of C-S-H chains and the relative populations of associated silicate species are determined, which can be used to evaluate the validity of molecular models for Portland cement hydration that have been previously proposed in the literature^17^,^21^,^47^. This opens new perspective for understanding the complex molecular-level mechanical properties of C-S-H.

Solid-state ^29^Si NMR measurements of ^29^Si-enriched triclinic Ca_3_SiO_5_ also enable the transient silicate speciation and polymerization in the developing C-S-H structure to be monitored and quantified as a function of hydration time, especially during the crucial induction, acceleration and deceleration stages. Importantly, hydroxylated monomeric (Q^0^(h)) silicate species can be detected and quantified by using ^29^Si{^1^H} CPMAS NMR measurements to monitor changes in surface composition with the progress of hydration. The NMR results presented here establish that non-hydrated Ca_3_SiO_5_ particle surfaces predominantly consist of hydroxylated Q^0^ silicate species with negligible quantities of Q^1^ and Q^2^ hydration products, including for the pre-induction and induction stages of the hydration process. Such detailed insights of silicate-water mixtures have heretofore been challenging and often infeasible to determine by other characterization techniques due to the low absolute quantities, complicated structures and poor long-range order of the hydroxylated surface species. Compared with the induction period, the onset of silicate polymerization (that is, Q^1^ and/or Q^2^ species) during hydration corresponds to the formation of dimeric units in C-S-H during the acceleration stage, consistent with previous cement literature. Interestingly, during the deceleration stage the hydration rate reduces (at a hydration level of 50%) before any significant reduction of the Q^0^(h) populations are observed at the Ca_3_SiO_5_ surface. This corresponds to a relatively fast decrease in the reaction rate compared with the rate of reduction of the hydroxylated species available for reaction at the surface, which indicates that part of the surface is likely covered by C-S-H products. These results are consistent with previous studies that suggest that the rate of hydration is controlled by the surface coverage of C-S-H species during the deceleration stage^37^. Calculations based on a shrinking core model (hydration reaction slows down due to consumption of the particles) indicate that for monodispersed spherical particles, a decrease in volume by a factor of 0.5 would be accompanied by a decrease in surface area by a factor 0.63 (2^−2/3^). Ca_3_SiO_5_ particles are neither spherical nor monodisperse but the present NMR results are definitely not compatible with a shrinking core model. Consequently, the surface area available for reaction is clearly modified by the surface roughness produced by dissolution driven etching of the surface^38^.

The relations directly observed here for the first time between surface passivation and etching phenomena on the one hand and the succession of the induction, acceleration and deceleration stages of hydration of Ca_3_SiO_5_ on the other hand, provide new understanding for the occurrence of this complex kinetic behaviour actually observed in a variety of silicate systems. Ca_3_SiO_5_, because of its high reactivity, constitutes an interesting model for understanding long term silicate hydration processes occurring during geochemical weathering or hydrothermal synthesis^23^.

## Methods

### NMR spectroscopy

The ^1^H and ^29^Si NMR isotropic chemical shifts were referenced to tetramethylsilane using tetrakis(trimethylsilyl)silane [((CH_3_)_3_Si_4_)Si] as a secondary standard^52^. All measurements were performed using zirconia MAS rotors and at room temperature. Solid-state 1D ^29^Si NMR experiments were carried out using a Bruker Avance-III 500 spectrometer (magnetic field 11.7 T). Magic-angle-spinning (MAS) spectra were measured using a Bruker MAS NMR probe with 4 mm rotors, at spinning frequencies of 7 kHz, and without decoupling. The single-pulse ^29^Si MAS NMR spectra were acquired with a *π*/2 pulse length of 6 μs, a recycle delay of 1,000 or 100 s, and 64 or 16 scans for the ^29^Si-enriched non-hydrated and hydrated Ca_3_SiO_5_ samples, respectively. {^1^H}^29^Si CPMAS NMR spectra were recorded using a ^1^H rf power of 93 kHz, a contact time of 5 ms, and recycle delay of 10 s. The number of scans was 184 for hydrated Ca_3_SiO_5_ samples and 2,000 for non-hydrated sample. Hartmann–Hahn matching was ensured by a ramp on the ^29^Si rf field intensity. 2D {^1^H}-^29^Si heteronuclear dipolar correlation (HETCOR) experiments were conducted on a Bruker Avance-700 (16.4 T) spectrometer at ambient temperature, under 4 kHz MAS conditions, with a 7 ms CP contact time, recycle delay of 10 s and 66 *t*_1_ increments of 50 μs each. Solid-state 2D *J*-mediated ^29^Si{^29^Si} DQ correlation NMR experiments were conducted using the refocused-INADEQUATE technique^39^ and a 18.8 T Bruker AVANCE-III NMR spectrometer. The experiments were conducted under conditions of 12.5 kHz MAS using a Bruker 3.2 mm H-X double resonance probehead. The 2D ^29^Si{^29^Si} spectrum was acquired using a 2.5 μs ^1^H *π*/2 pulse, 3.5 ms contact time for ^29^Si{^1^H} CP, 6.0 μs ^29^Si *π*/2 pulses, SPINAL-64 ^1^H decoupling^53^, 152 *t*_1_ increments, an incremental step size of 80 μs, a recycle delay of 2 s and 3,072 scans for each *t*_1_ increment, which corresponds to an experimental time of 260 h (∼11 days).

### Hydration experiments

Paste for *in situ* NMR measurements was prepared by mixing 0.3 g of non-hydrated ^29^Si-enriched Ca_3_SiO_5_ and 0.24 g of ultrapure water in a cylindrical 2 ml plastic vial for 3 min using a vortex mixer (Analog, VWR) at 2,500 r.p.m. With the help of a syringe and needle, part this paste was introduced as such in the zirconia MAS rotor thus enabling the acquisition of the NMR spectra during the reaction and avoiding any possible microstructural changes caused by the commonly used drying techniques^24^. After 6 h of hydration, the paste was removed from the ZrO_2_ rotor to prevent its hardening inside the rotor, and the NMR measurements were continued on the part of the sample previously set aside and stored in the closed vial at room temperature. The kinetics of ^29^Si-enriched Ca_3_SiO_5_ hydration were measured by isothermal calorimetry using a TAM Air microcalorimeter at 23 °C. One gram of ^29^Si-enriched Ca_3_SiO_5_ was mixed with 0.8 g of ultrapure water under identical conditions as for samples prepared for NMR measurements. The paste was immediately sealed in a glass ampoule and placed in the isothermal calorimeter. The degree of reaction of ^29^Si-enriched Ca_3_SiO_5_ was calculated by dividing the cumulative heat released at a certain time by the enthalpy of the hydration reaction of Ca_3_SiO_5_ (−520 J g^−1^ Ca_3_SiO_5_) (refs 54, 55). Additional details of synthesis, Ca_3_SiO_5_ characterization and NMR quantitative analysis are reported in the Supplementary Methods and Supplementary Note 1.

## Additional information

**How to cite this article:** Pustovgar, E. *et al*. Understanding silicate hydration from quantitative analyses of hydrating tricalcium silicates. *Nat. Commun.* 7:10952 doi: 10.1038/ncomms10952 (2016).

## Supplementary Material

Supplementary Information Supplementary Figures 1-12, Supplementary Tables 1-2, Supplementary Notes 1-4, Supplementary Methods and Supplementary References.
