# Supplementary material for: Understanding silicate hydration from quantitative analyses of hydrating tricalcium silicates
Source: Nat Commun. 2016 Mar 24;7:10952. doi: 10.1038/ncomms10952 (PMC4820784; doi:10.1038/ncomms10952)
Supplement: Supplementary Information — Supplementary Figures 1-12, Supplementary Tables 1-2, Supplementary Notes 1-4, Supplementary Methods and Supplementary References. [file ncomms10952-s1.pdf]

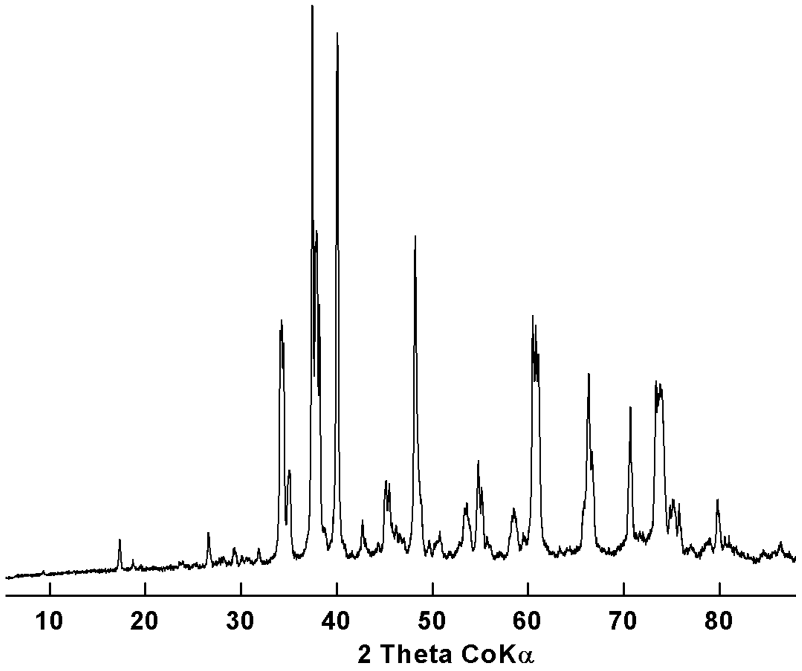

Supplementary Figure 1. XRD pattern of as-synthesized pure triclinic ^{29}Si-enriched Ca_3SiO_5.

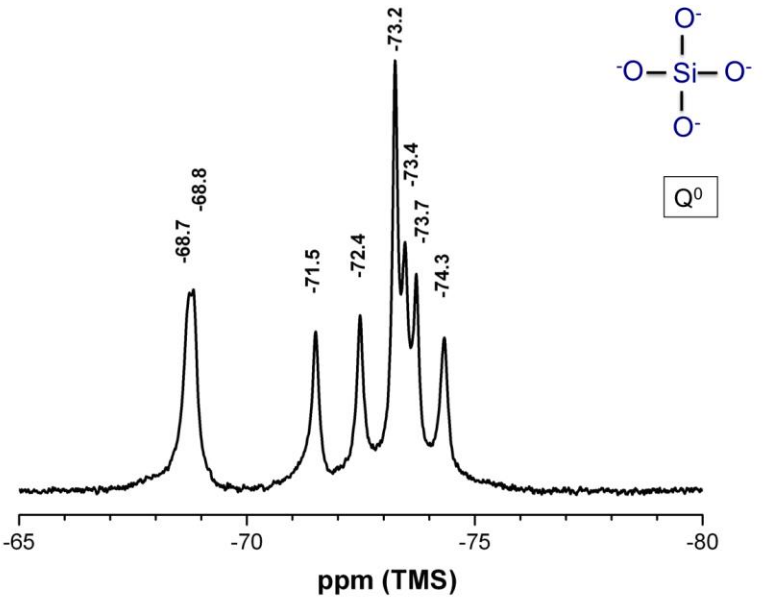

Supplementary Figure 2. Solid-state ^{29}Si MAS NMR spectrum of as-synthesized ^{29}Si-enriched Ca_3SiO_5.

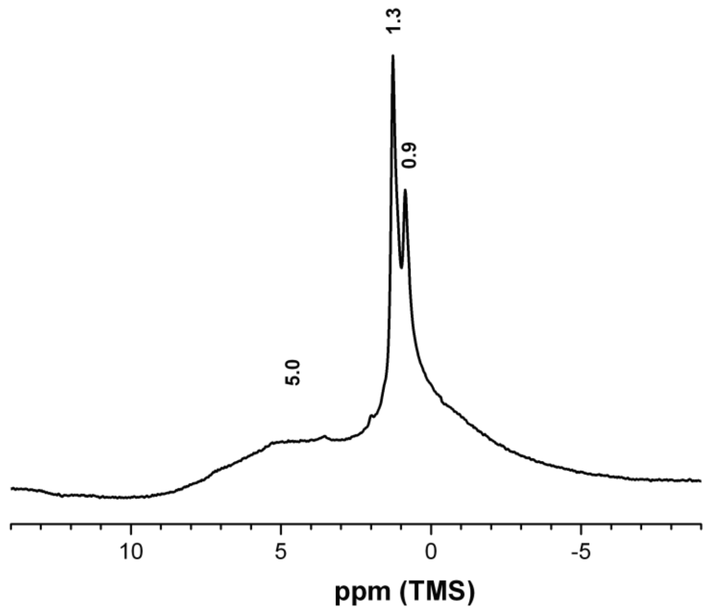

Supplementary Figure 3. Solid-state ^{1}H MAS NMR spectrum of as-synthesized (before hydration experiments) ^{29}Si-enriched Ca_3SiO_5.

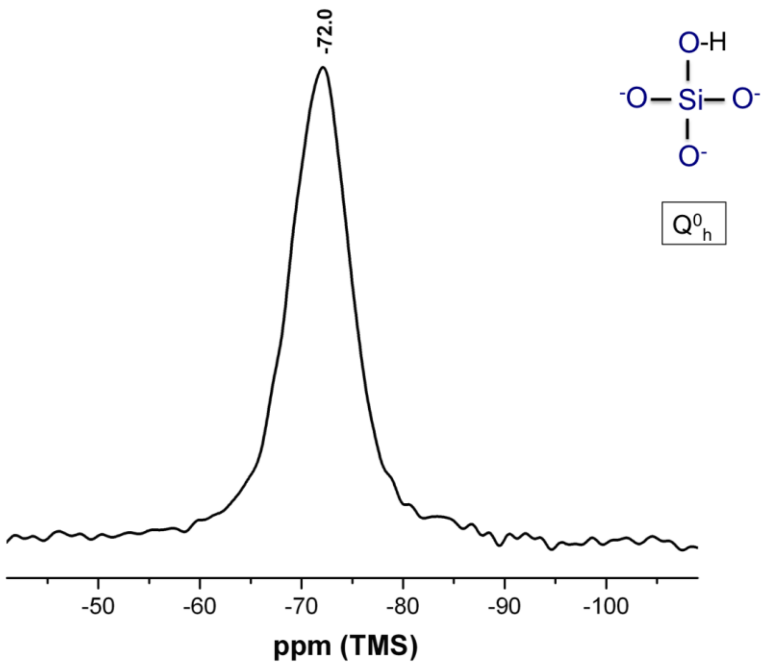

Supplementary Figure 4. Solid-state ^{1}H – ^{29}Si CPMAS NMR spectrum of as-synthesized (before hydration experiment) ^{29}Si-enriched Ca_3SiO_5.

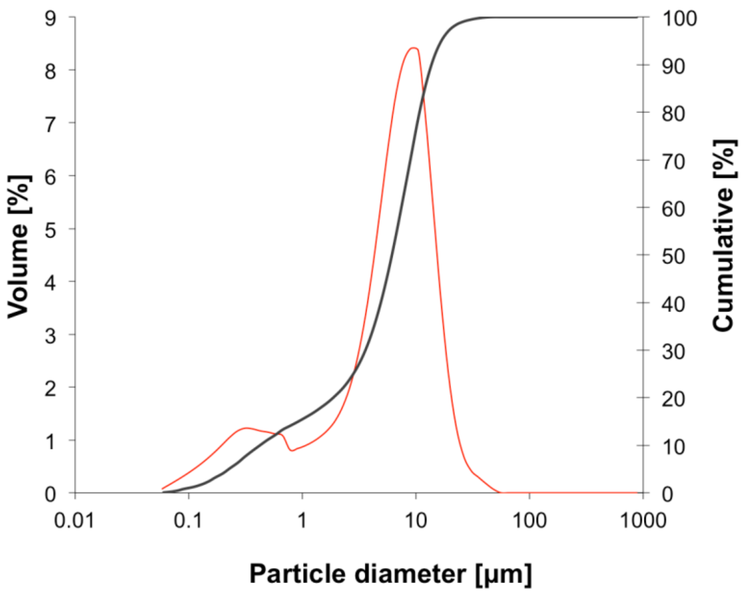

Supplementary Figure 5. Particle size distribution of as-synthesized ^{29}Si-enriched Ca_3SiO_5.

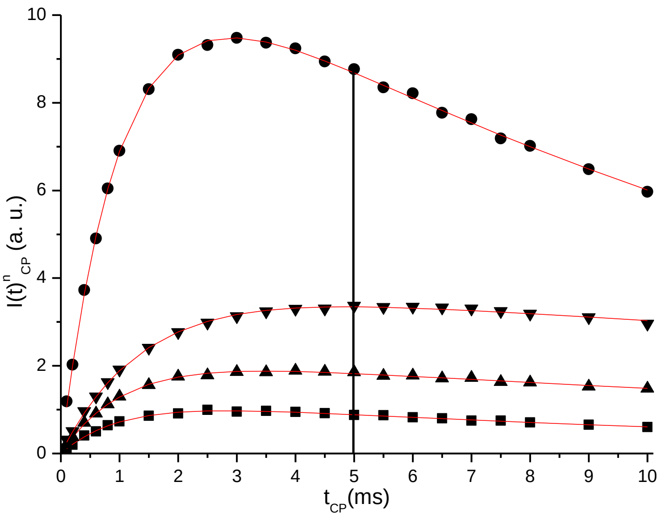

Supplementary Figure 6. Polarization growth curve on Ca_3SiO_5 hydrated for 90 days. The bar marks the 5 ms contact time used to measure $I^n_{cp}(t)$ and from which the quantitative intensities $I^n_{cp}(t)'$ are derived. The continuous curves are fits to Supplementary Equation (7).

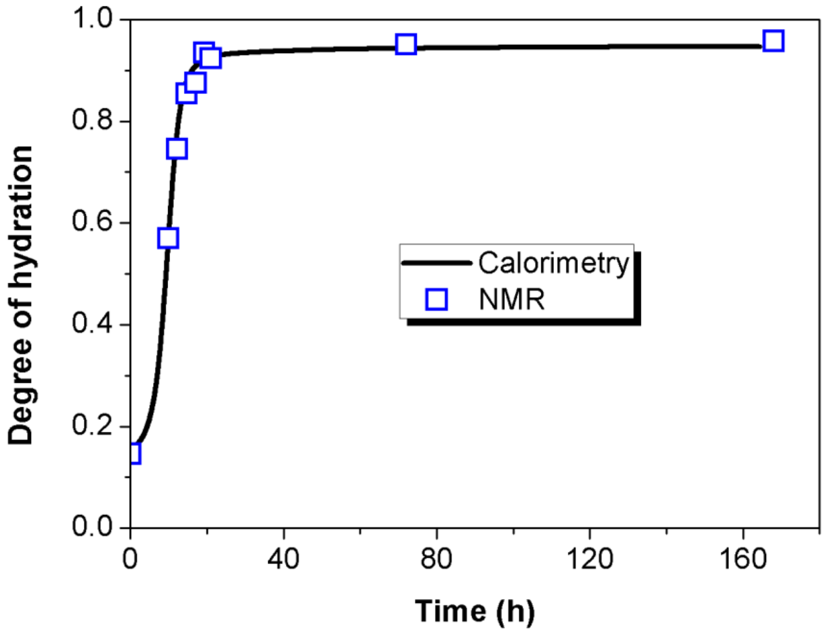

Supplementary Figure 7. Comparison of the degree of reaction calculated from quantitative NMR results (in red) and isothermal calorimetry (in blue).

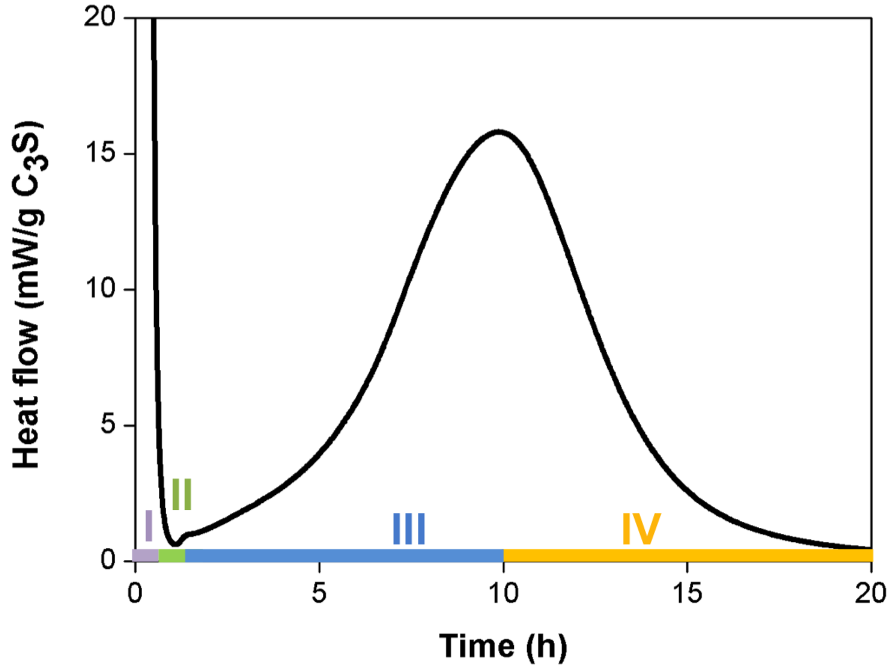

Supplementary Figure 8. Isothermal calorimetry curve of Ca_3SiO_5 hydration. Four regions are typically identified based on the heat flow profile with increasing hydration time, including pre-induction (I), induction (II), acceleration (III), and deceleration (IV) stages.

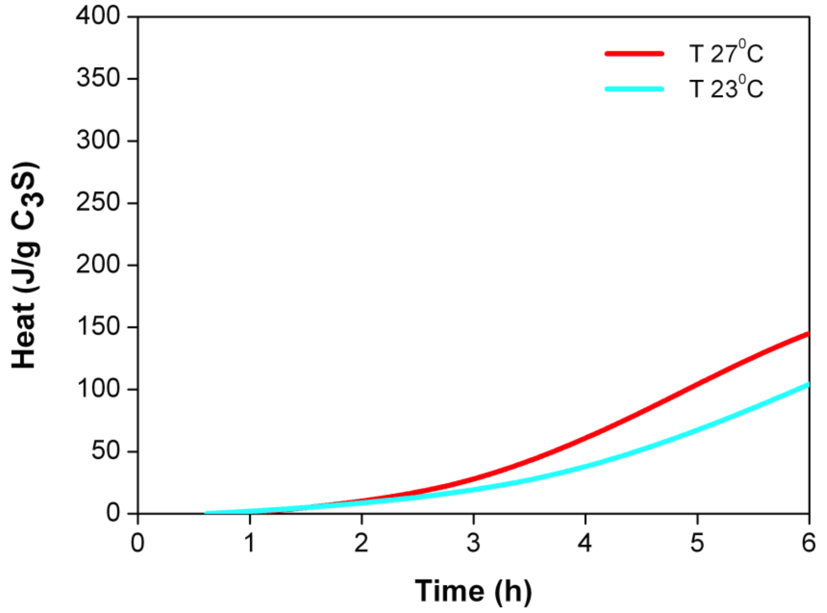

Supplementary Figure 9. Hydration of Ca_3SiO_5 at 23 and 27 ^{0}C studied by isothermal calorimetry.

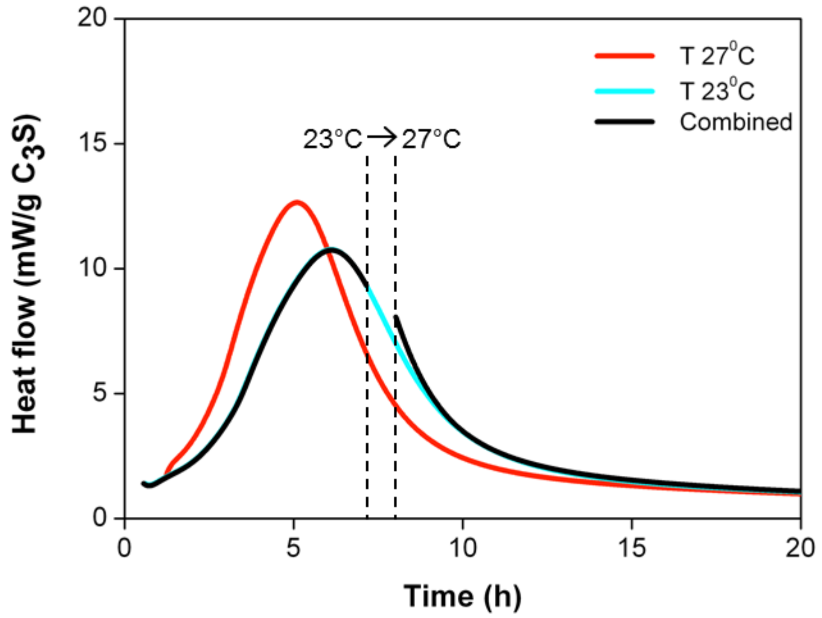

Supplementary Figure 10. Heat release rate during hydration of Ca_3SiO_5 studied by isothermal calorimetry at 23 ^{0}C (cyan), 27 ^{0}C (red) and under a combined cycle of 7 h at 23 ^{0}C followed by an increase to 27 ^{0}C (black). The gap corresponds the time needed to switch the sample between the calorimeters and to stabilize the equipment.

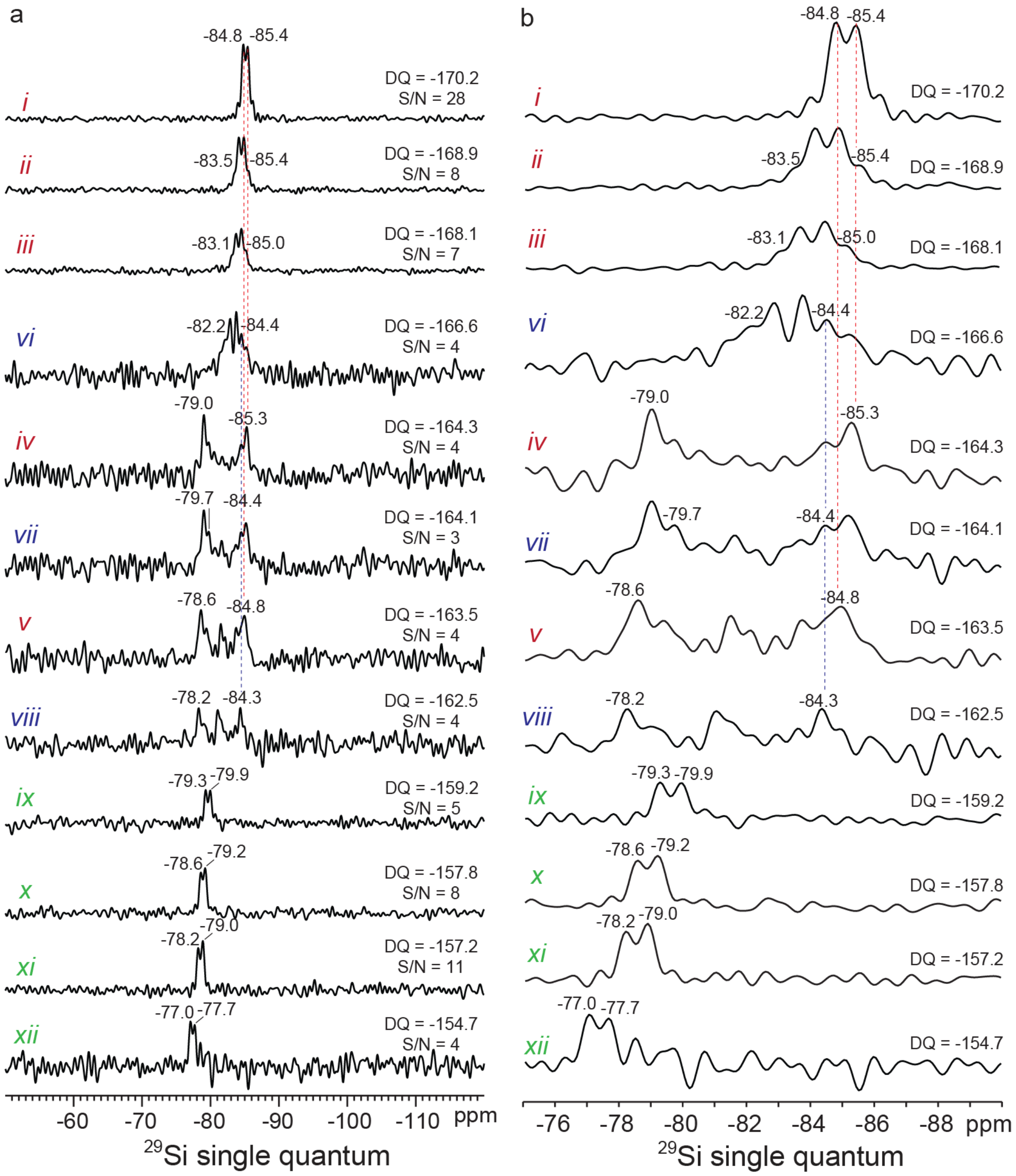

Supplementary Figure 11. Site connectivity in C-S-H. a,b, 1D ^{29}Si spectra that are slices at different double-quantum (DQ) ^{29}Si chemical shifts from the 2D *J*-mediated ^{29}Si{^{29}Si} correlation NMR spectrum (Supplementary Figure 4b). Different pairs of intensity correlations (labelled by roman numerals in the 2D spectrum) between ^{29}Si signals at distinct frequencies (ω_i, ω_j) in the single-quantum (SQ) dimension (isotropic ^{29}Si chemical shifts) and at the sum of these frequencies ($\omega_i + \omega_j$) in the DQ dimension (indicated for each slice) unambiguously establish that the associated distinct ^{29}Si species (i, j) are covalently bonded

through a shared bridging oxygen atom. The spectra in (**b**) are identical to those in (**a**), but shown over a narrower single-quantum ^{29}Si chemical shift range (-75 to -90 ppm). The DQ ^{29}Si chemical shifts and signal-to-noise ratios (S/N) are indicated for each slice.

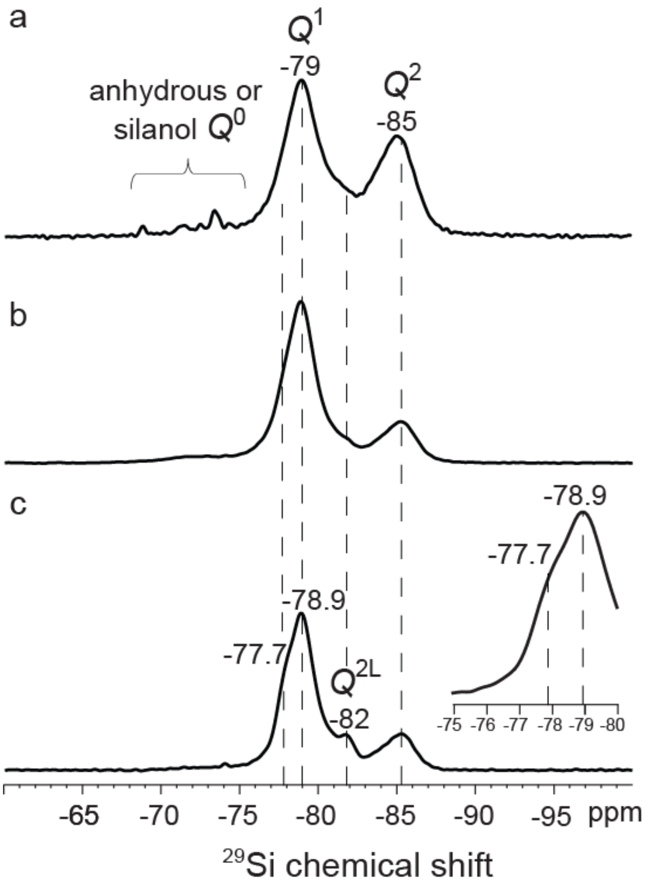

Supplementary Figure 12. Different extents of silicate polymerization in C-S-H. **a-c**, Solid-state 1D (**a**) single-pulse ^{29}Si, (**b**) ^{29}Si{^{1}H} cross-polarization (CP), and (**c**) T_2-filtered ^{29}Si{^{1}H} CP MAS spectra of hydrated (1.5 month, 25 °C) ^{29}Si-enriched triclinic Ca_3SiO_5. The spectra were acquired at (**a**) 18.8 T, 12.5 kHz MAS, 25 °C or (**b, c**) 11.7 T, 4.6 kHz MAS, 25 ºC. In (**c**), a nuclear spin-spin (T_2) relaxation-time filter (t_{T2}) of 20 ms and a ^{29}Si 180° pulse were applied before signal detection, to detect selectively ^{29}Si species with long (>20 ms) T_2 relaxation times. The inset in (**c**) shows a magnified version of the spectrum over the chemical shift range of -75 to -80 ppm.

Supplementary Table 1. Fit parameters for each type of ^{29}Si environments in hydrated Ca_3SiO_5.

One-pulse spectra										
chemical environment	Q^0							Q^1	Q^{2L}	Q^2
chemical shift (ppm)	-68.82	-71.55	-72.53	-73.29	-73.52	-73.77	-74.36	-78.7	-81.5	-85.2
width (ppm)	0.3	0.23	0.2	0.17	0.18	0.17	0.18	2.1	2.74	2.37
xG/(1-x)L	0	0	0	0	0	0	0	0.1	0.79	1
^1H - ^{29}Si cross polarization spectra										
chemical environment	Q^0(h)							Q^1	Q^{2L}	Q^2
chemical shift (ppm)	-72.1							-78.7	-81.5	-85.2
width (ppm)	6.84							2.44	1.3	2.73
xG/(1-x)L	0.68							0.1	0.79	1

SupplementaryTable 2. Average measured $\bar{k}^n$ values.

$\bar{k}^1$	$\bar{k}^2$
1.06 ± 0.02	0.94 ± 0.25

Supplementary Note 1. Quantitative comparison between isothermal calorimetry and NMR

Quantitative analysis of the NMR spectra.

The originality of the NMR results presented here lies mainly in their quantitative analysis. This is not trivial as cross-polarisation experiments are known to provide signal intensities that depend on several parameters, namely the Hartman-Hahn matching, the relaxation rates, the dipolar coupling, molecular mobility, and the extent of the proton bath. It is thus important to provide in detail the procedure followed that allowed seamless quantification based on the cross-polarisation and one-pulse experiments.

All one-pulse NMR experiments were performed with identical acquisition conditions. The same is true for the cross polarisation experiments (see Methods).

From the NMR spectra in the frequency domain, the integrated intensity corresponding to each chemical environment Q^n, was determined by decomposition of the ^{29}Si one-pulse and ^{1}H-^{29}Si CP MAS spectra using the program DMFit 2011.[3] To fit the signals a mixture of Gaussian (G) and Lorentzian (L) functions was applied as a model, which was kept constant together with the position and width for each specific resonance throughout the analyses of the NMR spectra (Supplementary Table 1).

After all integrated intensities were obtained, they were normalized to the number of scans, receiver gain and mass of Ca_3SiO_5 used for the measurements. The normalized intensities obtained by one-pulse and cross-polarization at a given time t are noted $I_{op}^{n}(t)$ and $I_{cp}^{n}(t)$ respectively.

Because a fully relaxed one-pulse NMR signal is directly proportional to the number of exited spins (here ^{29}Si) and because the number of ^{29}Si atoms introduced in the rotor is constant for a given hydration experiment, $I_{op}^{n}(t)$ can be expressed directly in arbitrary units of atoms of Si per mass of Ca_3SiO_5.

In contrast, the cross-polarization NMR spectra are affected by the spins relaxation and polarization transfer dynamics.[4] In samples with natural abundance ^{29}Si, the heat capacity of the silicon spin systems can be neglected compared to the one of the protons. Considering further that the relaxation of the silicon nuclei is much slower than the one of the protons, the cross-polarization dynamics between the ^{29}Si and the ^{1}H can be modelled as the superimposition of two first-order kinetics, namely the one of the ^{1}H relaxation in the rotating frame and the one of the polarization transfers between each ^{29}Si species and a unique ^{1}H bath.[5] In ^{29}Si enriched samples however, the assumption that the ^{29}Si spin heat capacities can be neglected versus the one of the ^{1}H falls. Consequently, there is no analytical solution to the set of differential equations describing the polarization intensity as a function of the contact time and the only option is to assess the relative heat capacities of each silicon spin bath by numerical modelling, as discussed by I. Klur in his doctoral thesis.[6] To avoid this lengthy procedure, it was preferable to calibrate the cross polarization response at a given contact time with respect to the quantitative one-pulse response. The following procedure was followed.

At a given contact time (5 ms), $I_{cp}^{n}(t)$ is measured (note that *t* is the hydration time, not the contact time of the cross polarization experiment) (Supplementary Figure 6). $I_{cp}^{n}(t)$ is proportional to the number of ^{29}Si spins but the proportionality constant *(k^n)* between the quantitative one pulse and the cross polarization intensities for the chemical environment Q^n,

$$k^{n} = \frac{I_{op}^{n}\left(t\right)}{I_{cp}^{n}\left(t\right)}, \qquad (2)$$

is an unknown function of the experimental conditions (Hartman-Hahn mismatch, rotation speed, contact time). It strongly depends on the acquisition conditions of the cross polarization spectra but will be a constant for a given chemical environment if these conditions are not varied as is the case in the present study. k^{n} is thus obtained by

comparison of the cross polarization intensities, $I_{cp}^{n}(t)$, with the quantitative one pulse ones, $I_{op}^{n}(t)$, at hydration times when both experiments provide a satisfactory signal to noise ratio. In practice, $k^{n}\left(t\right)$ is evaluated for hydration time *t* 21 h, 72 h, 168 h and 672 h, and the average $\bar{k}^{n}$ is calculated. The values are reported in the Supplementary Table 2.

At shorter time when only the cross polarization spectra can be obtained, this allowed to estimate a quantitative cross-polarization "one pulse-like" intensity, $I_{cp}^{n}(t)'$, by inverting Supplementary Equation (2)

$$I_{cp}^{n}(t)' = \bar{k}^{n} \times I_{cp}^{n}(t) \tag{3}$$

Since the $Q^0(h)$ species is a minor occurrence not revealed with sufficient resolution by one-pulse NMR, $\bar{k}^{0(h)}$ is not measurable in that manner. It was thus assumed that the true proportion of the $Q^0(h)$ resonance in the cross-polarization spectra was close to the one in the one-pulse spectra (which is equivalent to make the approximation of a common $\bar{k}^{n}$ value for all silicon species)

$$\frac{I_{cp}^{0(h)}\left(t\right)'}{I_{cp}^{1}\left(t\right)'+I_{cp}^{2}\left(t\right)'} = \frac{I_{cp}^{0(h)}\left(t\right)}{I_{cp}^{1}\left(t\right)+I_{cp}^{2}\left(t\right)} \tag{4}$$

and thus that $I_{cp}^{0(h)}\left(t\right)'$ can be estimated from the one-pulse and cross-polarization spectra decomposition through

$$I_{cp}^{0(h)}\left(t\right)' = \left[\bar{k}^{1} I_{cp}^{1}\left(t\right) + \bar{k}^{2} I_{cp}^{2}\left(t\right)\right] \frac{I_{cp}^{0(h)}\left(t\right)}{I_{cp}^{1}\left(t\right)+I_{cp}^{2}\left(t\right)} \tag{5}$$

When the Q^1 and Q^2 resonances where not observable in the cross polarization spectra (t < 1h), $I_{cp}^{0}\left(t<1h\right)'$ was obtained simply by comparing with the intensity at later time:

$$I_{cp}^{0(h)}\left(t\le 1h\right)' = I_{cp}^{0(h)}\left(t>1h\right)' \frac{I_{cp}^{0(h)}\left(t\le 1h\right)}{I_{cp}^{0(h)}\left(t>1h\right)} \tag{6}$$

This simplified procedure is an approximation as in principle, the polarisation transfer efficiency, and thus the conversion coefficient k is expected to differ for each Q^n resonance. This approximation was validated in the following manner. On a selected sample (hydration time 90 days), the quantitative intensity was extracted from a full polarisation signal build-up curve (Supplementary Figure 6), neglecting the heat capacity of the silicon spin bath using the usual polarization growth relation and adjusting the polarization transfer and relaxation parameters T_{CP} and $T_{1\rho}{}^H$

$$I_{cp}^n(t) = I_{cp}^n(t)'\alpha \frac{1}{1 - T_{cp}/T_{1\rho}^H}\left[\exp\left(-t_{cp}/T_{1\rho}^H\right) - \exp\left(-t_{cp}/T_{cp}\right)\right] \tag{7}$$

where the Hartman-Hahn mismatch α only acts as a constant scaling factor.

The resulting relative intensities were found to be within 5% of the ones obtained by our simplified procedure.

Finally, $Q^n(t)$, the proportion of silicon atoms in each Q^n environment in arbitrary units at hydration time t is obtained from the cross polarization spectra through

$$Q^n(t) = I_{cp}^n(t)' = \bar{k}^n \times I_{cp}^n(t) \tag{8}$$

for hydration times up to 10 h,

and from the one-pulse spectra directly through

$$Q^n(t) = I_{op}^n(t) \tag{9}$$

for longer hydration times.

Validation of the NMR quantitative analysis by comparison with isothermal calorimetry.

The degree of reaction (α) was calculated based on NMR quantitative analyses and compared with the value obtained by calorimetric measurement (Supplementary Figure 7). The values based on NMR data were calculated according to the following equations:

$$\alpha = 1 - \frac{Q^0(t)}{Q^0(t=0)} \tag{10}$$

and

$$\alpha = \frac{Q^{0(h)}\left(t\right) + Q^{1}\left(t\right) + Q^{2}\left(t\right)}{Q^{0}\left(t = 0\right)} \tag{11}$$

The NMR data matched the calorimetry results thus establishing the validity of the NMR quantitative analysis.

Supplementary Note 2. Isothermal calorimetry

The reaction of Ca_3SiO_5 with water is exothermic and, according to isothermal calorimetry, four main stages can be distinguished in the reaction of Ca_3SiO_5 with water (Supplementary Figure 8). The first one, the pre-induction period, is normally attributed to a rapid hydrolysis of the Ca_3SiO_5 surface releasing Ca^{2+}, OH^- and $H_2SiO_4^{2-}$ ions into solution. This is followed by a step with low reaction rate called the induction period (II), the origin of which has so far been poorly understood. Large amounts of C-S-H and $Ca(OH)_2$ are precipitated during the subsequent acceleration period (III), where nucleation and growth of the hydration products is the rate-controlling step. Finally, during the deceleration period (IV), C-S-H densification results in a decrease in the rate of silicate dissolution.

Since the sample temperature in the in-situ NMR measurements may deviate from ”room temperature” as a result of frictional heating during MAS experiments, calorimetric measurements were performed to assess potential frictional heating artefacts (Supplementary Figure 9). Indeed, this effect has been evaluated to account for a 4 K increase under our experimental conditions using the lead nitrate procedure.[7,8] Following our protocol for the NMR measurements, after 6 h of hydration the paste was removed from the ZrO_2 rotor to prevent its hardening inside the rotor and the subsequent loss of the rotor. The NMR measurements were continued on the part of the sample previously set aside in the closed vial and stored at room temperature.

The maximum possible consequences of a 4 K temperature variation are illustrated with the isothermal calorimetry measurements represented in Supplementary Figures 9 and 10. These were run with Ca_3SiO_5 paste at 27 and 23°C. The first three hours are identical, with a difference emerging only between 3 and 6 hours. In fact, this explains the slight observed difference at that time in the comparison between NMR and calorimetry data of Figure 2c. The cumulative effect of this temperature variation was appreciated by measuring the heat

release of the paste at 23°C for the first 7 h and then moving the sample to another calorimeter set at 27°C for the remaining experimental time. The heat flow (and thus the advancement of hydration) obtained during this measurement is very close to the one observed at 23°C. It can thus be concluded that the effect of the frictional heating during MAS is negligible under our experimental conditions.

Supplementary Note 3. Final C-S-H structure

The quantitative single-pulse ^{29}Si MAS spectrum (Supplementary Figure 12a) reveals signals from all anhydrous and hydrated ^{29}Si species, including Q^0, Q^1, and Q^2 moieties, as previously discussed in the main text of the manuscript. In contrast, the ^{29}Si{^{1}H} CPMAS experiment selectively detects ^{29}Si nuclei that are dipole-dipole-coupled to ^{1}H nuclei and thus are in close (<1 nm) molecular-level proximity. Correspondingly, the ^{29}Si{^{1}H} CPMAS spectrum exhibits ^{29}Si signals centred at -73 ppm (from Q^0(h) species), -79 ppm (from hydrated Q^1 species), and -85 ppm (from hydrated Q^2 species) that arise from ^{29}Si species in silicate hydration products. The relative intensities of the ^{29}Si signals in the ^{29}Si{^{1}H} CPMAS spectrum depend on the strengths of the heteronuclear ^{29}Si-^{1}H dipole-dipole couplings, which are determined by the molecular proximities and mobility of the associated chemical species. Nevertheless, signals with similar isotropic ^{29}Si chemical shifts, but with different relative intensities, are observed in the spectra in Supplementary Figure 12a,b.

By comparison, improved spectral resolution can be achieved by exploiting differences in the nuclear spin-spin (T_2) relaxation times of different hydrated silicate species to resolve their otherwise overlapping ^{29}Si signals. The T_2 relaxation times are sensitive to molecular structures and proximities of ^{29}Si species to dipole-dipole-coupled species, including ^{1}H moieties, and consequently can be used to distinguish their associated ^{29}Si NMR signals. This is accomplished by applying a T_2-filter (t_{T2}), during which signals from fast-relaxing ^{29}Si species ($T_2 < t_{T2}$) decay away, followed by a ^{29}Si 180° pulse (Hahn-echo-like technique) prior to the ^{29}Si NMR signal detection. The resulting signal consists of contributions exclusively from slow relaxing ($T_2 > t_{T2}$) ^{29}Si species in the calcium-silicate-hydrates. The calcium-silicate-hydrates exhibit strong through-space dipole-dipole-couplings between ^{29}Si and ^{1}H nuclei due to the abundance of ^{1}H species, including from hydroxyl moieties and water molecules, which are present in close (<1 nm) molecular proximity of the silicate

species. The magnitude of the T_2 relaxation time is attenuated by the increased strengths of the dipolar couplings, which consequently depend on molecular proximities and mobility. In ^{29}Si-enriched solids, as in the present case, the ^{29}Si-^{1}H dipole-dipole-couplings are further strengthened due to the significantly greater fractions of ^{29}Si nuclei compared to natural abundance conditions (4.7%), which correspondingly reduce the ^{29}Si T_2 relaxation times. These molecular-level structural differences are manifest in Supplementary Figure 12c as resolved ^{29}Si signals from the distinct silicate species, which were previously indistinguishable (Supplementary Figure 12a,b). For example, the T_2-filtered $^{29}Si\{^{1}H\}$ CPMAS spectrum exhibits partially resolved ^{29}Si signals at -77.7 and -78.9 ppm from chemically distinct Q^1 species that are associated with long T_2 relaxation times (>20 ms). Such structural differences in the ^{29}Si environments of Q^1 species that result in long T_2 relaxation times arise from their different proximities to water molecules and hydroxyl moieties, which influence the strengths of the heteronuclear ^{29}Si-^{1}H dipolar couplings. The ^{29}Si signal at -77.7 ppm (from Q^1 species) is consistent with the presence of dimeric C-S-H units that exhibit similar isotropic chemical shifts, as established based on the 2D *J*-mediated $^{29}Si\{^{29}Si\}$ spectrum (Fig. 5b). Additional ^{29}Si signal intensity is resolved at ca. 82 ppm and is attributed to Q^{2L} species. These results corroborate and complement the analyses of the 2D $^{29}Si\{^{29}Si\}$ spectrum, thereby increasing the confidence associated with the signal assignments and analysis of the silicate site connectivity.

Supplementary Note 4. Additional commentary on 2D *J*-mediated ^{29}Si{^{29}Si} NMR spectrum in Fig. 4b

Analyses of the pair correlated intensities in 2D ^{29}Si{^{29}Si} NMR spectrum of hydrated (1.5 month, 25 ºC) ^{29}Si-enriched Ca_3SiO_5 shown in Fig. 4b indicates the presence of pentameric C-S-H units, in addition to the octameric and dimeric units discussed in the main text of the manuscript.

A pentameric C-S-H unit consists of covalently bonded species with the sequence Q^1-Q^2-Q^{2L}-Q^2-Q^1 and thus does not present -Q^2-Q^2- connectivity. The latter can be observed only in an octamer Q^1-Q^2-Q^{2L}-$\mathbf{Q^2}$-$\mathbf{Q^2}$-Q^{2L}-Q^2-Q^1 or in longer chains. (Q^{2L}, the so-called bridging tetrahedral, are four-coordinate Q^2 silicate moieties that are positioned away from the interlayer space between C-S-H chains and link dimers, as shown in the inset in Fig. 4.) A series of connectivities including Q^1-Q^2, Q^2-Q^{2L} but no Q^2-Q^2 connectivities is thus a signature of pentamers. The 2D *J*-mediated ^{29}Si{^{29}Si} NMR spectrum in Fig. 4b reveals correlated intensities between the ^{29}Si SQ signal at -84.4 ppm from Q^2 species and -82.2 ppm (DQ = -166.6 ppm, *vi*), -79.7 ppm (DQ = -164.1 ppm, *vii*), and -78.2 ppm (DQ = -162.5 ppm, *viii*) that are associated with Q^{2L} species and two distinct Q^1 species, respectively. Therefore, the pairs of correlated intensities labelled *vi*, *vii*, and *viii* in the 2D spectrum (Fig. 4b) establish the presence of one Q^2-Q^{2L} and two distinct Q^2-Q^1 connectivity, respectively, in the associated C-S-H unit. Furthermore, there is no detectable correlated intensity, within the sensitivity limits of the measurement, between this Q^2 species with a ^{29}Si SQ signal at -84.4 ppm and any other resonance in the Q^2 range (only with Q^{2L} and Q^1 as stated above). This indicates that the associated C-S-H unit exhibits no direct Q^2-Q^2 connectivity, and thus, must be attributed to pentamers, as shown in the inset of Fig. 5b. This is in agreement with the previously proposed relative stabilities of C-S-H units of different chain lengths.[9,10]

Supplementary Methods

Synthesis of ^{29}Si-enriched Ca_3SiO_5

Pure tricalcium silicate (Ca_3SiO_5) with ^{29}Si isotopic enrichment was synthesized by the solid state reaction method:

$$3\ CaCO_3 + SiO_2 \longrightarrow Ca_3SiO_5 + CO_2\uparrow \qquad (1)$$

Precursors $CaCO_3$ (≥99 %, Sigma Aldrich) and SiO_2 (99.9 % enriched in ^{29}Si, Cortecnet) in 2.94 : 1 mass ratio were dry homogenized in a turbula for 24 h. Batches of approximately half a gram of the resulting powder were pressed into 1 cm pellets under a 25 kN load and fired afterwards in a platinum crucible at 1600 °C for 8 h. Since the triclinic Ca_3SiO_5 is a metastable phase, which tends to dissociate into CaO and Ca_2SiO_4,[1] pellets were quenched by fast cooling under an air gun. To reach the full transformation of the starting materials into the desired triclinic phase, the whole procedure was repeated 4 times, until no reflections due to free lime (CaO) were observed by X-ray diffraction.

Loss of SiO_2 during the homogenization process was unavoidable due to the strong electrostatic attraction of SiO_2 particles to the walls of the plastic container. Consequently, deviation from the stoichiometric ratio was necessary to prevent CaO from remaining in the final material.

The final material was ground using an agate mortar and then with absolute ethanol in a micronizing mill (McCrone) with agate grinding elements for 10 min. Absolute ethanol was used to ensure a better dispersion of the particles and to prevent heating that may otherwise result in undesirable structural modifications of the Ca_3SiO_5.

Characterization

^{29}Si-enriched Ca_3SiO_5 was characterized by X-ray diffraction (Supplementary Figure 1) using a Bruker AXS D8 diffractometer (Co K_α, λ = 1.7903 Å). 2θ was scanned between 4 and 90° by steps of 0.02°. The X-ray tube was operated at 40 kV and 30 mA. Rietveld analyses were performed using the AutoQuan software and confirmed the triclinic crystal

structure of the final material establishing the absence of any significant amounts of impurities with long-range structural order (refinement factor “weighted pattern” R_{wp} = 8.7 %).

The ^{29}Si-enriched Ca_3SiO_5 was also characterized by solid-state nuclear magnetic resonance (NMR) using ^{29}Si, ^{1}H one-pulse and ^{1}H-^{29}Si cross-polarization (CP) experiments. The ^{29}Si one-pulse NMR spectrum (Figure S2) shows 8 ^{29}Si resonances corresponding to the 9 distinct crystallographically inequivalent Si-sites in triclinic Ca_3SiO_5 (two overlapping ^{29}Si resonances are observed at -73.2 ppm)**.**[2] With the help of ^{1}H one-pulse and ^{1}H-^{29}Si CP MAS NMR, it was possible to retrieve information concerning the initial state of the sample. It was found out that the material adsorbed water either during storage or milling since peaks at 0.9 ppm (-Ca-O-H), 1.3 ppm (-Si-O-H) and 5.0 ppm (H_2O) were observed in the ^{1}H NMR spectrum (Supplementary Figure 3). The ^{1}H – ^{29}Si CP NMR spectrum exhibited a signal centered at -72 ppm, which confirmed the presence of Q^0(h) (hydroxylated) species, even before mixing with water (Supplementary Figure 4).

The specific particle size distribution (Supplementary Figure 5) was measured by laser diffraction (Mastersizer, Malvern Instruments) after dispersion of the ^{29}Si-enriched Ca_3SiO_5 in isopropanol.

The surface area of the final Ca_3SiO_5 powder was 4.38 ± 0.02 m^2 / g. It was measured using a BET multi-point physisorption apparatus (Micromeritics Tristar II 3020). The sample was degassed in an external degassing station (VacPrep 061 from Micromeritics) at 200 °C for 1 h.

Supplementary references

1. Wesselsky, A. & Jensen, O. M. Synthesis of pure Portland cement phases. *Cem. Concr. Res.* **39,** 973 – 980 (2009).

2. Skibsted, J., Hjorth, J., & Jakobsen, H. J. Correlation between ^{29}Si NMR chemical shifts and mean Si-O bond lengths for calcium silicates. *Chem. Phys. Lett.* **172,** 279–283 (1990).

3. Massiot, D. et al. Modelling one and two-dimensional solid-state NMR spectra. *Magn. Reson. Chem.* **40,** 70 – 76 (2002).

4. Skibsted, J., Hjorth, L. & Jakobsen, H. J. Quantification of thaumasite in cementitious materials by ^{29}Si {$_1$H} cross-polarization magic-angle spinning NMR spectroscopy. *Adv. in Cem. Res*. **7,** 69-83 (1995).

5. Klur, I. Etude par RMN de la structure des silicates de calcium hydrates, PhD thesis, Université Paris VI, Paris, France (1996).

6. Klur, I. et al. NMR Cross-polarization when $T_{IS} > T_{1\rho}$; Examples from silica gel and calcium silicate hydrates. *J. Phys. Chem.* **104**, 10162-10167 (2000).

7. Grimmer, A. R., Kretschmer, A., Cajipe, V. B. Influence of Magic Angle Spinning on Sample Temperature. *Magn. Reson. Chem.* **35,** 86 - 90 (1997).

8. d'Espinose de Lacaillerie, J.-B., Jarry, B.,Pascui, O. &Reichert, D. "Cooking the sample": radiofrequency induced heating during solid-state NMR experiments. *Solid State Nucl. Magn. Reson.* **28**, 225-232 (2005).

9. Ayuela, A. et al. Silicate chain formation in the nanostructure of cement-based materials. *J. Chem. Phys.* **127,** 164710 (2007).

10. Brough, A. R., Dobson, C. M., Richardson, I. G. & Groves, G. W. In situ solid-state NMR studies of Ca_3SiO_5: hydration at room temperature and at elevated temperatures using ^{29}Si enrichment. *J. Mater. Sci.* **29,** 3926–3940 (1994).
